# Physical therapist-delivered motivational interviewing and health-related behaviour change: A systematic review and meta-analysis

**DOI:** 10.1016/j.bjpt.2024.101168

**Published:** 2024-12-31

**Authors:** Elizabeth Wintle, Nicholas F Taylor, Katherine Harding, Paul O'Halloran, Casey L Peiris

**Affiliations:** a Community Rehabilitation Program, Eastern Health, Melbourne, Victoria, Australia; b La Trobe University Academic and Research Collaborative in Health (ARCH), Melbourne, Australia; c Eastern Health Allied Health Clinical Research Office, Box Hill, Australia; d School of Psychology and Public Health La Trobe University, La Trobe University, Melbourne, Australia; e Allied Health, The Royal Melbourne Hospital, Parkville, Australia

**Keywords:** Exercise, Motivational interviewing, Physical therapy modalities, Rehabilitation

## Abstract

**Background:**

Motivational interviewing (MI) promotes health-related behaviour change and improves patient health outcomes, but the effect of physical therapist-delivered MI is unclear.

**Objective:**

To evaluate the effect of physical therapist-delivered MI on health-related behaviour change in adults attending physical therapy or rehabilitation.

**Methods:**

CINAHL, Medline, PubMed, PEDro, Embase, and Cochrane databases were searched in August 2023. Randomised controlled trials evaluating physical therapist-delivered MI and health-related behaviour change were included. Internal validity was evaluated using the PEDro scale. GRADE approach was used for each meta-analysis.

**Results:**

Ten publications from nine randomised controlled trials involving 909 participants were included. Physical therapist-delivered MI likely increased physical activity slightly (SMD 0.21, 95 % CI -0.05, 0.47) when compared to minimal intervention; however the evidence is very uncertain in relation to self-efficacy (SMD 0.51, 95 % CI -0.35, 1.38) and health-related quality of life (SMD 0.73, 95 % CI -0.64, 2.11). When physical therapist-delivered MI was combined with and compared to rehabilitation, there were no additional effects on physical activity (SMD 0.02, 95 % CI -0.37, 0.41), health-related quality of life (SMD 0.18, 95 % CI -0.27, 0.63), or endurance (SMD 0.15, 95 % CI -0.21, 0.52) and a likely small effect on self-efficacy (SMD 0.23, 95 % CI -0.1, 0.55).

**Conclusion:**

Physical therapist-delivered MI is likely to produce a small improvement in physical activity, but only in the absence of other comprehensive rehabilitation. The most beneficial application of MI may be for patients who are not receiving rehabilitation or who have low levels of motivation and self-efficacy.

## Highlights

• Physical therapists can proficiently deliver motivational interviewing (MI)
• MI produced a small increase in physical activity compared to minimal intervention.
• Usual-care physical therapy may sufficiently address health-related behaviour change.
• MI may be most beneficial for patients not actively receiving physical therapy care.

## Introduction

As primary care practitioners specialising in prevention and management strategies, physical therapists are well positioned to align physical therapy practice with global health priorities towards adopting and sustaining positive health behaviours.^1^ The prescription of therapeutic exercise and tailored education, hallmarks of physical therapy practice, are well established interventions to promote positive health behaviour change and improve patient health outcomes.^2^ However, the efficacy of these interventions rely on patient adherence and the extent to which a patient is actively engaged with their therapy. While physical therapists traditionally rely on education to facilitate behaviour change, it is unclear how to best deliver education to improve patient health outcomes.^3^ Poor adherence to treatment advices remains widespread within the patient population^4^ suggestive that education alone is not sufficient to bridge the intention-behaviour gap.^5^

It has been proposed that initiating and sustaining behaviour change is not supported through the provision of expert advice alone, but rather through empowering individuals by enhancing their intrinsic motivation for behaviour change.^6^ Motivational interviewing (MI) is an evidence-based patient-centred counselling approach that can bridge the gap between prescription and adherence by exploring and resolving ambivalence.^6^ In this way, MI seeks to enhance an individual's intrinsic motivation for change towards positive health behaviours. Though originally developed in the addiction field and delivered by psychology-based professionals, MI has since been applied across a broad range of clinical populations to address several health behaviours. Of particular relevance to physical therapy practice is its ability to improve physical activity behaviour^7^, ^8^ and adherence to health recommendations.^9^ Several positive patient health outcomes have also been demonstrated including improved health-related quality of life (QoL) and self-efficacy,^9^ improved symptom management,^10^ and reduced hospital admissions.^9^

Despite evidence to support the use of MI to facilitate outcomes in physical therapy, the efficacy of physical therapist-delivered MI has not been explored systematically. MI proficiency appears not to be correlated with the professional background of the therapist delivering the MI,^11^^,^^12^ **r**ather, (two-day workshop) led by a trainer accredited by the Motivational Interviewing Network of Trainers (MINT) coupled with individual feedback and coaching.^13^ With training, physical therapists could potentially embed behavioural counselling methods such as MI into their daily practice. Despite this, MI is often considered as an adjunct to physical therapy, delivered by other trained health professionals. One systematic review investigated the addition of motivational interventions to physical therapy, but interventions were not limited to MI and were delivered by a physical therapist in only 3 of the 14 trials.^14^ Several reviews exploring the role of MI and physical activity, arguably a core domain of physical therapy, include trials where MI was delivered by counsellors, researchers, and nurses.^7^^,^^8^^,^^15^

This review aims to determine the effectiveness of MI delivered by physical therapists on behaviour change and patient health outcomes compared to usual care or alternate intervention.

## Methods

This systematic review and meta-analysis is reported in accordance with the Preferred Reporting Items for Systematic Reviews and Meta-analysis guidelines^16^ and was registered prospectively (March 2023) with PROSPERO (CRD42023408220).

### Information sources and search strategy

A systematic literature search was conducted from inception until August 2023 in six databases: CINAHL complete (EBSCO), Medline (Ovid), PubMed central (PMC), Physiotherapy Evidence Database (PEDro), Embase (Ovid), and the Cochrane library (Central). The search terms and strategies were developed and piloted in consultation with the research team and a specialist health science librarian.

Keywords for motivational interviewing, physiotherapy/physical therapy and rehabilitation were mapped to medical subject headings and combined with keyword search results (including truncations to account for variations in spelling). Results for ‘physical therapy’/ ‘physiotherapy’ “OR” ‘rehabilitation’ were combined with results for ‘motivational interviewing’ using the “AND” operator (see Supplementary material for full search strategies).

### Eligibility criteria

The inclusion criteria followed the PICOs framework (population, intervention, comparator, outcome, study design).^17^ Studies were included if they were peer-reviewed randomised controlled trials evaluating physical therapist-delivered MI focussed on health-related behaviour change (e.g. physical activity, dietary intake) in adults attending physical therapy / rehabilitation (including physical therapy) with or without co-intervention/usual care (full inclusion criteria provided in Supplementary material).

Trials evaluating MI for mental health conditions and/or addiction were excluded. Trials where MI was combined with other behavioural interventions, e.g. cognitive behavioural therapy, were excluded to enable this review to focus on the behavioural effect of MI. Studies that used motivational enhancement therapy, a brief therapeutic approach using MI principles, were also excluded.

### Selection process

After removal of duplicates, two reviewers independently screened titles and abstracts and then the remaining full-text articles for inclusion via the online platform Covidence.^18^ Agreement between reviewers was recorded. Disagreements between reviewers were resolved by a third reviewer, or through discussion within the team. Reference lists of included papers were screened to identify any additional papers for review. Authors of full-text papers were contacted to clarify if physical therapists provided the MI intervention if this was not clear.

### Data items and collection process

Data extraction was completed independently by two reviewers (EW, CP). Data were extracted into a customised excel spreadsheet and included demographic factors (age, sex, country, year of study, health condition, setting), experimental and control interventions, primary (e.g. physical activity) and secondary outcomes (patient health outcomes) and time points, adverse events, and results. Because MI efficacy is associated with treatment fidelity,^19^ MI treatment fidelity and methods to confirm proficiency of the MI physical therapists were also extracted. Where the required information was not clearly reported, lead authors of included papers were contacted to request additional detail.

### Study risk of bias assessment

Internal validity of included trials were evaluated using the Physiotherapy Evidence Database (PEDro) scale; a valid, unidimensional measure of methodological quality of clinical trials.^20^ The PEDro scale has demonstrated ‘fair’ to ‘excellent’ inter-rater reliability for physical therapy-related clinical trials,^21^ and evidence of construct and convergent validity.^22^ Studies scoring < 4 are considered to demonstrate ‘poor’ methodological quality, 4 to 5 considered ‘fair’, 6 to 8 are considered ‘good’, and 9 to 10 are considered ‘excellent’.^23^ For studies evaluating complex interventions such as MI, a PEDro score of 8/10 is often the highest score obtainable as blinding of therapists is not possible and blinding of participants is challenging.^20^^,^^21^

Two reviewers (EW and NT or KH) independently applied the tool, and disagreements between reviewers were resolved through discussion or by a third reviewer (CP) as necessary. Agreement between reviewers was recorded.

### Data analysis

Standardised mean differences (SMD) and 95 % confidence intervals (CI) were calculated from pooled post-intervention means and standard deviations (SD). Where results were not reported as means and SDs, they were manually converted according to recommendations.^24^ Clinically homogeneous data were synthesised using random effects models for outcomes using Revman software (Review Manager version 5.4).^25^ Strength of the SMD was reported according to Cohen^26^ where 0.2 represents a small effect, 0.5 a moderate effect, and 0.8 a large effect.^26^ Trial results were synthesised for common outcomes including physical activity, health-related QoL, self-efficacy, and endurance (e.g. 6 min walk test [MWT]). If a trial reported several different measures of an outcome (e.g. physical activity), the common measure between the trials was used. Statistical heterogeneity was assessed using the I^2^ statistic with significant heterogeneity defined as I^2^ >50 %.^27^

### Certainty assessment

The GRADE (Grading of Recommendations, Assessment, Development, and Evaluations) approach was applied to determine the certainty of evidence for each meta-analysis.^28^ The GRADE approach considers four levels of certainty (very low to high) where randomised controlled trials begin with a high certainty and are downgraded if there are concerns about risk of bias, inconsistency, indirectness, imprecision, and publication bias.

Evidence was downgraded by one level: if there were concerns about the methodological quality (PEDro < 6)^23^ of the majority of trials (i.e. >50 %) (risk of bias); if the statistical test for heterogeneity demonstrated substantial heterogeneity (I^2^ >50 %)^29^ (inconsistency); if there were significant concerns that the participants, intervention, comparator, or outcome were inconsistent with usual clinical practice (e.g. participants who wouldn't usually see a physical therapist) or if there was evidence of indirect comparisons (e.g. participants who are seeing a physical therapist vs healthy controls)^30^ (indirectness); if the 95 % CI of the SMD was wide (i.e. >0.8) indicating imprecision (such that the lower band of the 95 % CI could indicate little or no effect while the upper band could indicate a large effect)^26^ or if the CI crossed the null effect threshold^31^ (imprecision); or if publication bias was strongly suspected, for example if the analysis included mainly small studies showing statistically significant results or inclusion of trials with industry influence.^32^ Evidence was downgraded two places if both criteria for imprecision were met. Single trials were considered inconsistent and imprecise, thereby providing low certainty evidence. This could be further downgraded to very low certainty evidence if there was high risk of bias.

Results and certainty of the evidence have been reported according to the GRADE guidelines on informative statements to communicate the findings of systematic reviews.^33^

## Results

### Study selection

**Fig. 1:**
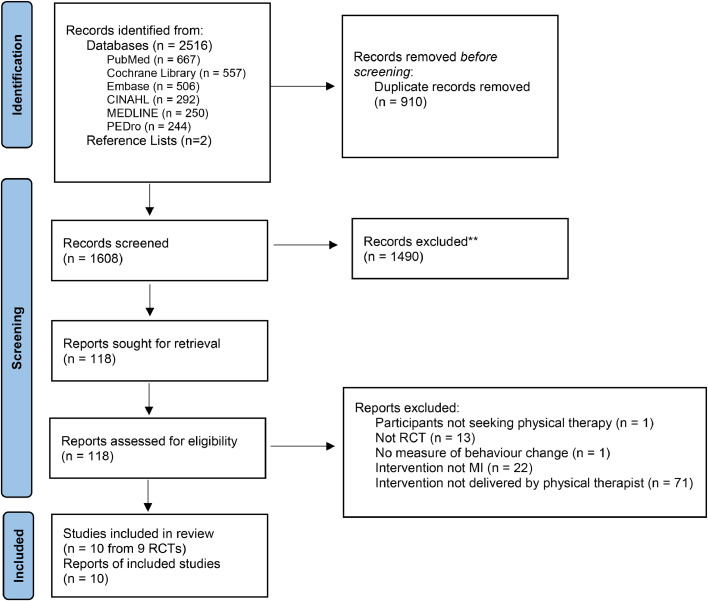
Flow of trials through the review. MI, motivational interviewing; RCT, randomised controlled trial.

After duplicates were removed, 1608 articles were screened on title and abstract, of which 118 underwent full text screening. After excluding 108 ineligible articles (Supplementary material, full-text exclusions), 10 publications from 9 independent randomised controlled trials met the final eligibility criteria for inclusion (Fig. 1). Findings from one trial were reported in two separate papers, one reporting initial findings^34^ and the other reporting 12-month follow up data.^35^ For the purpose of this review, these publications will collectively be referred to as the primary trial by Arkkukangas.^34^ Inter-rater agreement was ‘moderate’ at title and abstract screening (*k* = 0.44, 95 % CI 0.36, 0.52), and full text screening (*k* = 0.57, 95 % CI 0.34, 0.80). Two authors were contacted for the unadjusted post intervention data,^36^^,^^37^ and one provided further data.^37^

### Trial and participant characteristics

The nine randomised controlled trials were published between 2012 and 2022 (Table 1) and included 909 participants. Five trials were completed in Europe (Sweden, Belgium, Spain, Denmark, Switzerland),^34^^,^^36^^,^^38^, ^39^ two in Australia, ,^40^^,^^41^ one in Canada,^42^ and one in the USA.^37^

**Table 1:** Trial characteristics (*n* = 9).

Study, PEDro score	Clinical setting, Country	Population *n*= int:cont, diagnostic group, Mean (SD) age	Intervention	Control	Health related outcome/s and measures	Time points
Arbillaga-Etxarri et al.^38^ 6/10	33 primary care centres and 5 hospitals, Spain	*n* = 132:148 COPD 69 (8) years 244 males (87 %)	1 × 1 hour in person MI session followed by 1–4 × 5–10 min telephone MI (based on motivation level) + URBAN TRAINING - walking ≥ 5 days/week, pedometer monitoring, written education, text messages, walking group, phone support + Usual standard management (as per control)	Usual standard pharmacological and/or non-pharmacological management for COPD, including pulmonary rehab.	PA: accelerometer Health-related QoL Endurance	Baseline 52 weeks
Arkkukangas et al.^34^ and Tuvemo Johnson et al.^35^ 8/10	Community dwelling, Sweden	*n* = 58:61:56* Older community dwelling adults 83 (5) years 53 males (30 %)	MI embedded into 5 × 1 hour home visits over 12 weeks + OTAGO exercise program (as per control)	OTAGO exercise program: written information and home exercise program including balance, strength training, and walking.	PA: Frandin/Grimby activity scale Mobility, strength, balance, exercise, adherence, self-efficacy	Baseline 12 weeks 42 weeks
Burtin et al.^36^ 6/10	Pulmonary rehabilitation (hospital), Belgium	*n* = 40:40 COPD 66 (7) years 65 males (81 %)	8 × 20–30 min in person MI sessions over 6 months + Multidisciplinary pulmonary rehabilitation (as per control)	Multidisciplinary pulmonary rehabilitation: 3 x week for 3 months, then twice weekly for 3 months. + Sham attention program	PA: accelerometer	Baseline 12 weeks 26 weeks
Dennett et al.^40^ 8/10	Outpatient oncology rehab, Australia	*n* = 22:24 Cancer 59 (12) years 17 males (37 %)	7 x∼ 21-minute telephone MI delivered over 7 weeks + Oncology Rehabilitation (as per control)	Oncology Rehabilitation (twice weekly 7 weeks)	PA: accelerometer Health-related QoL Functional impairment Strength Endurance	Baseline 8 weeks
Larsen et al.^43^ 8/10	Community dwelling, Denmark	*n* = 32:38 Community dwelling older adults 72 (3) years 28 males (40 %)	7 x ∼18-minute telephone MI Delivered over 12 weeks + Usual care (as per control)	PA monitor and national PA recommendations	PA: accelerometer Health-related QoL Self-efficacy	Baseline 12 weeks
O'Halloran et al.^41^ 6/10	Community dwelling, Australia	*n* = 16:14 Post hip fracture 83 (5) years 5 males (17 %)	8 × 30-minute telephone MI delivered over 8 weeks + Usual care (as per control)	Usual care e.g. general practitioner visits, community physical therapy	PA: accelerometer Health-related QoL Mobility Self-efficacy	Baseline 9 weeks
Rausch Osthoff et al.^39^ 6/10	Pulmonary rehabilitation (hospital), Switzerland	*n* = 17:25 COPD 68 (8) years 21 males (50 %)	5 × 30-minute in person MI delivered over 12 weeks + Pulmonary rehabilitation (as per control)	Pulmonary rehabilitation - twice weekly over 12 weeks, weekly supervised Nordic walking	PA: accelerometer Health-related QoL Endurance	Baseline 12 weeks 24 weeks
Reid et al.^42^ 7/10	Cardiac centre, Canada	*n* = 69:72 Coronary artery disease 61 (10) years 103 males (73 %)	1 × 25–35-minute in person MI + 8 × 10–15-minute telephone MI delivered over 52 weeks + Usual care (as per control)	Cardiology discharge booklet, advice about the importance of physical activity	PA: pedometer	Baseline 26 weeks 52 weeks
Pellegrini et al.^37^ 5/10	Outpatient physical therapy, USA	*n* = 24:21 Post total knee replacement (TKR) 65 (7) years 18 males (40 %)	Approximately 12 x MI sessions embedded in usual care physical therapy 2–3 x week delivered over 5–6 weeks. + Usual care physical therapy following total knee replacement (as per control)	Usual care physical therapy following total knee replacement	PA: accelerometer Functional impairment Endurance Pain	Baseline 12 weeks

NB: *3 armed RCT – OTAGO exercise program +MI vs OTAGO exercise program vs control (minimal intervention).

COPD, chronic obstructive pulmonary disease; int:con, intervention:control; MI, motivational interviewing PA, physical activity; QoL: Quality of life; TKR, total knee replacement.

Three trials included participants with chronic obstructive pulmonary disease (COPD),^36^^,^^38^^,^^39^ two recruited older adults from the community,^34^^,^^43^ and single trials included participants with coronary artery disease,^42^ cancer,^40^ post hip fracture,^41^ and post total knee replacement.^37^ The mean age of participants ranged from 59^40^ to 83 years old^34^^,^^41^ and, overall, 61 % (554) were male (Table 1).

### Intervention characteristics

Physical therapist-delivered MI was added to usual care and compared to usual care alone in 8 of the 9 trials.^34^^,^^36^, ^37^^,^^42^^,^^43^ In three of these trials, usual care consisted of minimal intervention (such as written advice or routine general practitioner visits)^43^^,^^41^, ^42^ and in five trials, usual care consisted of comprehensive rehabilitation involving physical therapy with or without multi-disciplinary team care.^34^^,^^36^, ^37^^,^^39^, ^40^ In one of the trials where MI was delivered as an adjunct to comprehensive rehabilitation, a third group received minimal intervention.^34^

The ninth trial was the only one which included a co-intervention. This trial examined physical therapist-delivered MI with a structured walking program and usual care management compared with usual care management alone.^38^

### Mode of delivery of motivational interviewing

Motivational interviewing was delivered in person in four trials,^34^^,^^36^, ^37^^,^^39^ via telephone in three trials,^43^^,^^40^, ^41^ and in combination in two trials with MI initially delivered in person and subsequently delivered by telephone.^38^^,^^42^ Physical therapists delivered between two^38^ and 12^37^ MI sessions over an intervention period ranging from seven^40^ to 52 weeks (Table 1).^38^^,^^42^

### Training

Four trials demonstrated the minimum recommended training requirements for MI proficiency,^34^^,^^39^, ^40^, ^41^ including between two^40^^,^^41^ and nine^39^ days of training and feedback from a MINT trainer (Table 2). One trial provided individual training sessions by an experienced health psychologist,^36^ and one provided minimal MI training led by the principal investigator (exercise physiology background).^37^ Three studies did not report who provided the MI training.^38^, ^39^, ^40^, ^41^, ^42^, ^43^^,^^42^

**Table 2:** MI characteristics.

Study	MI training	Core components of MI reported	Fidelity measure and outcome
Arbillaga-Etxarri et al.^38^	Insufficient details provided to confirm minimal training requirements met	Relational component - spirit of MI Technical component (i.e. OARS)	Uncertain. Not confirmed with a validated MI fidelity measure: *Training sessions, support and supervision, periodic recording*
*Arkkukangas et al.^34^ and *Tuvemo Johnson et al.^35^	Training meets minimal requirements to achieve proficiency: *3-day training with 2 x MINT trainer, 3 booster sessions*	Relational component - spirit of MI Technical component (i.e. OARS) Underlying principles of MI present throughout.	Confirmed with a validated MI fidelity measure (MI coding lab)
Burtin et al.^36^	Does not meet minimal training requirements: *3×* 60 min *individual training sessions by an experienced health psychologist*	Relational component - spirit of MI Stage matched MI approach based on low/high scores of motivation (self-efficacy scale 0–10)	Uncertain. Not confirmed with a validated MI fidelity measure: *Interviews with patients video-taped and discussed with health psychologist in individual or group sessions in the first few months of the study*
Dennett et al.^40^	Training meets minimal requirements to achieve proficiency: *2-day workshop, online training, 1:1 coaching from a MINT trainer*	Relational component - spirit of MI Technical component (i.e. OARS)	Confirmed with a validated MI fidelity measure (MITI 4.1)
Larsen et al.^43^	Insufficient details provided to confirm minimal training requirements met: *4-day course with reading materials, discussion and role play exercises*	Relational component - spirit of MI Technical component (i.e. OARS)	Confirmed with a validated MI fidelity measure (MITI 4)
O'Halloran et al.^41^	Training meets minimal requirements to achieve proficiency: *2-day workshop, online training, 1:1 coaching from a MINT trainer*	Relational component - spirit of MI Technical component (i.e. OARS)	Confirmed with a validated MI fidelity measure (MITI 3.1.1)
Rausch Osthoff et al.^39^	Training meets minimal requirements to achieve proficiency: *9-day MI course with a MINT trainer with feedback to MI physios*	Relational component - spirit of MI Technical component (i.e. OARS)	Confirmed with a validated MI fidelity measure (MITI 4.2.1)
Reid et al.^42^	Insufficient details provided to confirm minimal training requirements met: *2-days training, regular case discussions*	MI processes - engage, evoke, action planning	Uncertain. Not confirmed with a validated MI fidelity measure: *Used scripts / checklists to maintain intervention fidelity*
Pellegrini et al.^37^	Does not meet minimal training requirements: *Brief training on techniques aligned with principles of MI led by the PI (EP background)*	Relational component - spirit of MI Technical components (i.e. OARS)	MI fidelity not confirmed: *59/377 (15 %) physical therapy sessions checked for fidelity with unannounced observations of physical therapy session by 5 reviewers*

Abbreviations: EP, exercise physiologist; MI, motivational interviewing; MINT, motivational interviewing network of trainers; MITI, motivational interviewing treatment integrity; OARS, open questioning, affirming, reflecting and summarising; PI, principal investigator.

### Fidelity

Four trials^39^, ^40^, ^41^^,^^43^ confirmed MI fidelity with the validated Motivational Interviewing Treatment Integrity (MITI) Scale.^44^ Of these, two confirmed proficiency via role play with a MINT accredited MI trainer prior to the study commencement.^40^, ^41^ Other fidelity measures included controlled coding during the study at a MI coding lab,^34^ unannounced observations by trained reviewers against a fidelity checklist,^37^ and review of audiotaped MI sessions by a health psychologist.^36^ Two trials reported the use of training, support, periodic supervision, scripts, and checklists to maintain intervention fidelity (Table 2).^38^^,^^42^

### Outcome measures

Eight trials measured the primary outcome of physical activity with an accelerometer or pedometer.^36^, ^37^, ^38^, ^39^, ^40^, ^41^, ^42^ Six reported daily steps,^36^^,^^38^, ^39^, ^40^, ^41^ and one reported kilometres travelled over seven days.^42^ Four trials reported minutes of moderate to vigorous physical activity (MVPA) using an accelerometer^37^^,^^40^ or a physical activity questionnaire.^43^^,^^42^ A single trial measured physical activity subjectively via a 6-point self-reported activity scale.^34^ Two trials evaluated physical activity at long-term follow-up.^35^^,^^39^ No included trials reported other behaviour change outcomes such as those related to diet or medication adherence.

Commonly reported secondary outcomes included health-related QoL (*n* = 5), self-efficacy (*n* = 4), and endurance (*n* = 5). Health-related QoL was measured using the Assessment of Quality of Life Instrument,^41^ European Organization for Research and Treatment of Cancer QoL Questionaire-C30,^40^ euroQol-5 Domain Quality of Life questionnaire,^43^ chronic respiratory disease questionnaire (CRQ),^39^ the clinical COPD questionnaire (CCQ),^38^ and as a subset of the Knee Injury and Osteoarthritis Outcome Score (KOOS).^37^ Self-efficacy was evaluated using the ambulatory self-confidence questionnaire,^41^ physical activity appraisal inventory,^40^ falls efficacy Swedish scale,^34^ and the self-efficacy for exercise scale.^43^ Endurance was measured via the 6MWT.^36–38,39–40.^

### Risk of bias in studies

The mean PEDro score of the included trials was 6.7 out of 10, ranging from 5^37^ to 8^34,^^43^^,^^40^ (Supplementary material, PEDro methodological quality assessment). Eight of the 9 trials demonstrated good methodological quality (PEDro score 6–8)^34^^,^^36^^,^^38^, ^39^, ^40^, ^41^^,^^42^ with the remaining study demonstrating fair methodological quality.^37^ All trials fulfilled random allocation, baseline comparability, reported between-group differences, and provided point estimates and estimates of variability. Eight trials used blinded assessors^34^^,^^36^^,^^38^, ^39^, ^40^, ^41^, ^42^ and five trials followed an intention-to-treat analysis.^34^^,^^38^, ^39^, ^40^, ^41^, ^42^, ^43^^,^^40^^,^^42^ There was almost perfect agreement between reviewers who assessed methodological quality (kappa 0.96, 95 % CI 0.90, 1.00).

### Effect of physical therapist-delivered motivational interviewing

#### MI with minimal intervention vs. minimal intervention alone

**Fig. 2:**
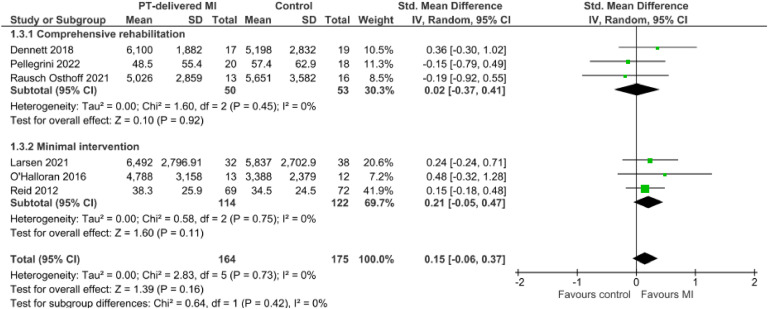
Meta-analysis of the effect of physical therapy-delivered motivational interviewing (MI) on physical activity.

Meta-analysis of 3 trials^41^, ^42^, ^43^ with 236 participants produced moderate certainty evidence that physical therapist-delivered MI likely results in a slight increase in physical activity when combined with minimal intervention and compared to minimal intervention alone (SMD 0.21, 95 % CI −0.05, 0.47, I^2^ 0 %) (Fig. 2, Table 3).

**Table 3:** Summary of findings and certainty of evidence GRADE.

Effect of physical therapist-delivered MI with minimal intervention vs. minimal intervention alone													
Certainty assessment							№ of patients		Effect		Certainty		Comments
№ of studies	Study design	Risk of bias	Inconsistency	Indirectness	Imprecision	Other considerations	Intervention	Control	Absolute (95 % CI)				
**Physical activity**													
3	randomised trials	not serious	not serious	not serious	serious^a^	none^b^	114	122	SMD 0.21 SD higher (0.05 lower to 0.47 higher)		⨁⨁⨁◯ Moderate		Physical therapist-delivered MI likely increases physical activity slightly when combined with and compared to minimal intervention
**Self efficacy**													
2	randomised trials	not serious	serious^c^	not serious	very serious^d^	none^b^	50	45	SMD 0.51 SD higher (0.35 lower to 1.38 higher)		⨁◯◯◯ Very low		Physical therapist-delivered MI may increase self-efficacy when combined with and compared to minimal intervention but the evidence is very uncertain
**Health related QoL**													
2	randomised trials	not serious	serious^c^	not serious	very serious^d^	none^b^	50	45	SMD 0.73 SD higher (0.64 lower to 2.11 higher)		⨁◯◯◯ Very low		Physical therapist-delivered MI may increase health-related quality of life when combined with and compared to minimal intervention but the evidence is very uncertain
Effect of physical therapist-delivered MI and comprehensive rehabilitation vs. comprehensive rehabilitation alone													
**Physical activity**													
3	randomised trials	not serious	not serious	not serious	serious^a^	none^b^	50	53	SMD 0.02 SD higher (0.37 lower to 0.41 higher)	⨁⨁⨁◯ Moderate		Physical therapist-delivered MI likely results in no difference in physical activity when combined with and compared to comprehensive rehabilitation	
**Self-efficacy**													
2	randomised trials	not serious	not serious	not serious	serious^a^	none^b^	71	77	SMD 0.23 SD higher (0.1 lower to 0.55 higher)	⨁⨁⨁◯ Moderate		Physical therapist-delivered MI likely increases self-efficacy slightly when combined with and compared to comprehensive rehabilitation	
**Health related QoL**													
2	randomised trials	not serious	not serious	not serious	very serious^c^	none^b^	34	44	SMD 0.18 SD higher (0.27 lower to 0.63 higher)	⨁⨁◯◯ Low		Physical therapist-delivered MI may result in little to no difference in health-related QoL when combined with and compared to comprehensive rehabilitation	
**6MWT**													
3	randomised trials	not serious	not serious	not serious	serious^a^	none^b^	53	64	SMD 0.15 SD higher (0.21 lower to 0.52 higher)	⨁⨁⨁◯ Moderate		Physical therapist-delivered MI likely results in little to no difference in endurance when combined with and compared to comprehensive rehabilitation	

CI**,** confidence interval; MI, motivational interviewing; QoL, quality of life; SMD**,** standardised mean difference.

Explanations.

a. Downgraded by 1 as the CI of the SMD includes the possibility of no effect.

b. Publication bias was undetected, though the included studies were small, no trials had industry influence and the included trials showed a range of positive and negative effects, therefore we did not downgrade for publication bias .

c. Downgraded by 1 due to substantial heterogeneity (I^2^ >50 %).

d. Downgraded by 2 as the CI of the SMD is wide (i.e. >0.8) indicating imprecision and includes the possibility of no effect.

CI**,** confidence interval; MI, motivational interviewing; QoL, quality of life; SMD**,** standardised mean difference; 6MWT, 6 min walk test.

Explanations.

a. Downgraded by 1 as the CI of the SMD includes the possibility of no effect.

b. Publication bias was undetected, though the included studies were small, no trials had industry influence and the included trials showed a range of positive and negative effects, therefore we did not downgrade for publication bias.

c. Downgraded by 2 as the CI of the SMD is wide (i.e. >0.8) indicating imprecision and includes the possibility of no effect.

For the secondary outcomes, meta-analyses of two trials^40^^,^ ^43^ demonstrated physical therapist-delivered MI may increase self-efficacy (SMD 0.51, 95 % CI −0.35, 1.38, I^2^ 69 %, 95 participants) and health-related QoL (SMD 0.73, 95 % CI −0.64, 2.11, I^2^ 86 %, 95 participants) when combined with and compared to minimal intervention, but the evidence is very uncertain (Table 3).

#### MI and comprehensive rehabilitation vs. comprehensive rehabilitation alone

Meta-analysis of 3 trials^37^^,^^39^^,^^40^ with 103 participants demonstrated that when combined with comprehensive rehabilitation, physical therapist-delivered MI likely results in no effect on physical activity (SMD 0.02, 95 % CI −0.37, 0.41, I^2^ 0 %) when compared to comprehensive rehabilitation alone (Fig. 2, Table 3).

Results from single trials that could not be included in the meta-analysis demonstrated little to no difference in physical activity between those who received MI with comprehensive rehabilitation and those who received comprehensive rehabilitation alone at the end of the intervention^34^^,^^36^ and at long term follow-up.^35^^,^^39^

For the secondary outcomes, meta-analyses of two trials,^34^,^40^ demonstrated physical therapist-delivered MI likely improves self-efficacy slightly (SMD 0.23, 95 % CI −0.10, 0.55, I^2^ 0 %, 148 participants) when combined with and compared to rehabilitation alone, but had little to no effect on health-related QoL^39^^,^^40^ (SMD 0.18, 95 % CI −0.27, 0.63, I^2^ 0 %, 78 participants) and endurance^37^^,^^39^^,^^40^ (6MWT) (SMD 0.15, 95 % CI −0.21, 0.52, I^2^ 0 %, 117 participants) (Table 3).

#### MI as a co-intervention with rehabilitation vs. usual care management

When MI was delivered as a co-intervention with rehabilitation (structured walking program) compared to usual care in a single trial with 280 participants,^38^ there was no effect on physical activity (SMD 0.04, 95 % CI −0.19, 0.28), health-related QoL (SMD 0.0, 95 % CI −0.24, 0.24), or endurance (6MWT) (SMD −0.05, 95 % CI −0.29, 0.19) at 12 months. However, the certainty of evidence from this single trial^38^ was low after downgrading for inconsistency and imprecision.

## Discussion

This review found that physical therapist-delivered MI likely improved physical activity slightly when compared to minimal intervention. When delivered with and compared to comprehensive rehabilitation, MI was unlikely to have an additional effect on physical activity. These findings are consistent with an overview of reviews that also found a small effect on behaviour change with MI, but adds to that review by taking into account the effect of the comparator intervention.^45^ The observed effect on physical activity (SMD 0.21) in the current review is similar to a previous review where the MI was mainly delivered by counsellors and educators (SMD 0.19).^7^ These results suggest that physical therapists, with modest training and support, can deliver MI proficiently, further supporting previous findings that MI fidelity is not related to the professional background of the treating therapist, rather on achieving the recommended training and support.^12^

It is possible that adding MI to usual physical therapy management may not result in improved health behaviour (physical activity) because physical therapists may already be promoting behaviour change through patient centred approaches towards achieving specific and meaningful goals. The theoretical model of expert practice in physical therapy^46^ encompasses many MI consistent principles; reflective listening, collaborative patient-centred care, and establishing mutual respect. It is possible that physical therapists delivering usual care rehabilitation may practice elements of MI through expert communication and establishing a collaborative therapeutic alliance.

Another possible explanation is that routine comprehensive rehabilitation programs were able to address and impact positive behaviour change through several mechanisms. While MI served to enhance the patient's intrinsic motivation for change, health behaviour is driven by complex interacting systems extending beyond motivation at the individual level, including the capability and opportunity for change.^47^ Usual-care rehabilitation involving physical therapy is likely to have a positive impact on the patient's physical capacity and provide the opportunity to engage in regular physical activity, hence promoting behaviour change. In this way, all participants in the intervention and control groups in these trials were receiving evidence-based behaviour change interventions regardless of whether they were allocated to the intervention or control groups. It follows that behavioural counselling like MI may be most indicated for patients for whom low motivation and low self-efficacy is the major barrier to behaviour change rather than physical capacity or opportunity.^48^^,^^49^

A positive finding was that physical therapists can proficiently implement MI as intended with relatively modest training and support. MI was administered both in person and via the telephone in an embedded and adjunct model, with trials confirming MI fidelity through validated and reliable MI integrity tools. This finding suggests that MI can be integrated into routine clinical practice in a variety of ways.

Considering the trivial to small effects on physical activity observed when MI is added to physical therapy interventions, a possible clinical implication of these findings is that MI may be most applicable in the absence of other robust physical therapy intervention. MI applied on discharge from in person rehabilitation may enable continued improvements in physical activity even when most supportive care has ceased.^41^ This approach may be particularly beneficial for patients where physical inactivity is predominantly driven by psychosocial rather than physical factors. Physical therapist-delivered MI may also enable an alternative option for patients who either cannot access or decline formal physical rehabilitation programs, where MI can be effectively delivered via telephone or telehealth.^42^

### Strengths and limitations

This review and meta-analysis has been conducted in accordance with the PRISMA reporting guidelines for systematic reviews and meta-analysis.^16^ All trials included in this review were peer reviewed randomised controlled trials, and predominantly good-quality trials. The GRADE approach^28^ was applied to each meta-analysis to evaluate the level of certainty of each finding. A particular strength of this review is reflected through the robust MI eligibility criteria ensuring only trials which confirmed MI fidelity with a validated and reliable MI integrity tool were included.

A potential limitation of this review is the low number of trials and limited sample size in each meta-analysis, which may have contributed to imprecision in estimates of effect. In addition, few trials included long-term follow-up. Finally, it is possible that through the robust eligibility criteria regarding MI treatment fidelity, some trials may not have been included due to reporting, rather than the quality of MI within the trial.

## Conclusion

This review indicates that physical therapists can proficiently deliver MI to likely improve physical activity by a small amount when compared to minimal intervention. Comprehensive physical therapy rehabilitation appears to sufficiently address health-related behaviour change for patients who are ready for change with no additional benefit of MI demonstrated. Future research should consider the application of MI by physical therapists for patients identified with low baseline levels of motivation and self-efficacy, and for those who are no longer supported by rehabilitation programs.

## Declaration of competing interest

The authors have no conflicts of competing interest to declare.

## Appendix Supplementary materials

Image, application 1
