# Supplementary material for: Physical therapist-delivered motivational interviewing and health-related behaviour change: A systematic review and meta-analysis
Source: Braz J Phys Ther. 2024 Dec 31;29(1):101168. doi: 10.1016/j.bjpt.2024.101168 (PMC11751405; doi:10.1016/j.bjpt.2024.101168)
Supplement: Supplementary file 1 [file mmc1.docx]

**Search strategies**

**Ovid - MEDLINE**

| 1. | Motivational interviewing/ |
| --- | --- |
| 2. | *motivational interviewing/ |
| 3. | motivation* interview*.mp. [mp=title, abstract, heading word, drug trade name, original title, device manufacturer, drug manufacturer, device trade name, keyword heading word, floating subheading word, candidate term word] |
| 4. | motivation* interview*.ti. |
| 5. | motivation* interview*.ab. |
| 6. | 1 or 2 or 3 or 4 or 5 |
| 7. | Physical Therapy Modalities/ |
| 8. | physiotherapy.tw. |
| 9. | physio therapy.tw. |
| 10. | physical therapy.tw. |
| 11. | physio.tw. |
| 12. | 7 or 8 or 9 or 10 or 11 |
| 13. | Rehabilitation Research/ or "Physical and Rehabilitation Medicine"/ or Stroke Rehabilitation/ or Neurological Rehabilitation/ or Hospitals, Rehabilitation/ or Rehabilitation Centers/ or Cardiac Rehabilitation/ or Rehabilitation/ |
| 14. | rehab*.tw. |
| 15. | 13 or 14 |
| 16. | 12 or 15 |
| 17. | 6 and 16 |

**Ovid - Embase**

| 1. | Motivational interviewing/ |
| --- | --- |
| 2. | *motivational interviewing/ |
| 3. | motivation* interview*.mp. [mp=title, abstract, heading word, drug trade name, original title, device manufacturer, drug manufacturer, device trade name, keyword heading word, floating subheading word, candidate term word] |
| 4. | motivation* interview*.ti. |
| 5. | motivation* interview*.ab. |
| 6. | 1 or 2 or 3 or 4 or 5 |
| 7. | Physical Therapy Modalities/ |
| 8. | physiotherapy.tw. |
| 9. | physical therapy.tw. |
| 10. | 7 or 8 or 9 |
| 10. | home rehabilitation/ or rehabilitation care/ or pulmonary rehabilitation/ or community based rehabilitation/ or rehabilitation center/ or rehabilitation patient/ or rehabilitation research/ or athletic rehabilitation/ or geriatric rehabilitation/ or rehabilitation/ or rehabilitation medicine/ or stroke rehabilitation/ or cancer rehabilitation/ or heart rehabilitation/ |
| 12. | rehab*.tw. |
| 13. | 11 or 12 |
| 14. | 10 or 13 |
| 15. | 6 and 14 |

.mp: free text searching in Ovid

**EBSCOhost – CINAHL**

| 1. | (MH "Motivational Interviewing") |
| --- | --- |
| 2. | 'motivational interviewing' |
| 3. | TI 'motivational interviewing' OR AB'motivational interviewing' |
| 4. | TI 'motivational interview' OR AB'motivational interview' |
| 5. | TI 'motivation interview*' OR AB'motivation interview*) |
| 6. | S1 OR S2 OR S3 OR S4 OR S5 |
| 7. | (MH "Physical Therapy") OR (MH"Home Physical Therapy") OR (MH"Chest Physical Therapy") OR (MH"Physical Therapy Practice") OR (MH"American Physical TherapyAssociation") OR (MH "PhysicalTherapy Practice, Research-Based")OR (MH "Physical Therapy Practice,Evidence-Based") OR (MH"Research, Physical Therapy") OR(MH "Education, Physical Therapy")OR (MH "Physical Therapy Service")OR (MH "Physical TherapyAssessment") |
| 8. | (MH "Physical Therapists") OR"physical therapist" OR (MH"Australian PhysiotherapyAssociation") OR (MH "PhysicalTherapy") OR "physiotherapy" |
| 9. | TI 'physio' OR AB 'physio' |
| 10. | TI 'physiotherapy' or AB'physiotherapy' |
| 10. | TI physical therapy' OR AB 'physicaltherapy' |
| 12. | \| S7 OR S8 OR S9 OR S10 OR S11 \| \| --- \| |
| 13. | \| (MH "Rehabilitation") OR (MH"Rehabilitation, Athletic") OR (MH"Rehabilitation Centers") OR (MH"Rehabilitation, Cancer") OR (MH"Rehabilitation, Pulmonary") OR (MH"Rehabilitation Patients") OR (MH"Home Rehabilitation") \| \| --- \| |
| 14. | \| TI 'rehab*' OR AB 'rehab*' \| \| --- \| |
| 15. | \| S13 OR S14 \| \| --- \| |
| 16. | \| S12 OR S15 \| \| --- \| |
| 17. | \| S6 AND S16 \| \| --- \| |

**Cochrane**

| 1. | MeSH descriptor: [Motivational Interviewing] explode all trees |
| --- | --- |
| 2. | (motivation* NEXT interview*):ti:ab:kw |
| 3. | #1 OR # 2 |
| 4. | MeSH descriptor: [Physical Therapy Modalities] explode all trees |
| 5. | MeSH descriptor: [Rehabilitation] explode all trees |
| 6. | (rehab*):ti:ab:kw |
| 7. | #5 OR #6 |
| 8. | #4 OR #7 |
| 9. | #3 AND # 8 |

**PEDro**

| 1. | motivation* interview*  limit to RCT |
| --- | --- |

**PubMed**

| 1. | motivational interviewing |
| --- | --- |
| 2. | physiotherapy |
| 3. | 'physical therapy' |
| 4. | (physiotherapy) OR ('physical therapy') |
| 5. | rehabilitation |
| 6. | rehab* |
| 7. | (rehabilitation) OR (rehab*) |
| 8. | rehabilitation[Title/Abstract] |
| 9. | rehab*[Title/Abstract] |
| 10. | (rehabilitation[Title/Abstract]) OR (rehab*[Title/Abstract]) |
| 11. | physiotherapy[Title/Abstract] |
| 12. | 'physical therapy'[Title/Abstract] |
| 13. | (physiotherapy[Title/Abstract]) OR ('physical therapy'[Title/Abstract]) |
| 14. | ((physiotherapy[Title/Abstract]) OR ('physical therapy'[Title/Abstract])) OR ((rehabilitation[Title/Abstract]) OR (rehab*[Title/Abstract])) |
| 15. | (((physiotherapy[Title/Abstract]) OR ('physical therapy'[Title/Abstract])) OR ((rehabilitation[Title/Abstract]) OR (rehab*[Title/Abstract]))) AND (motivational interviewing) |
| 16. | ((rehabilitation[Title/Abstract]) OR (rehab*[Title/Abstract])) OR ((physiotherapy) OR ('physical therapy')) |
| 17. | (((rehabilitation[Title/Abstract]) OR (rehab*[Title/Abstract])) OR ((physiotherapy) OR ('physical therapy'))) AND (motivational interviewing) |

**Inclusion criteria**

| **Population**   - Adults ≥ 18 years old seeking physical therapy or multidisciplinary rehabilitation (including physical therapy) for physical rehabilitation or health promotion. |
| --- |
| **Intervention**   - To be considered motivational interviewing (MI), the intervention had to report at least one of the following:   - The 3 core characteristics of MI: 1) a clear focus on changing behaviour; 2) use of empathic listening in a collaborative relationship to understand the person’s perspective about changing their behaviour; and 3) an emphasis on evoking the person’s motivation for change (i.e. person producing their own arguments for change) ^6^   - MI implementation fidelity (using a validated fidelity tool)   - MI training/proficiency of the physical therapists - MI delivered by physical therapists in any of the following circumstances:   - As a stand-alone intervention   - As an intervention within or alongside one or more co-interventions - As an intervention delivered as an adjunct to usual care rehabilitation or other usual care management - MI delivered in-person, via telephone or telehealth. App web-based MI were only included if used to complement physical therapist-delivered MI. |
| **Comparator**   - Treatment as usual/standard care without MI. - Placebo or control intervention e.g. sham MI or an alternative intervention to account for the additional attention received by the intervention group. - Alternate or minimal intervention without MI. |
| **Outcomes**   - Primary outcome: Health-related behaviour change outcome/s including but not limited to:   - Physical activity   - Adherence to health-related recommendations (e.g. medication adherence, diet changes)   - Self-management behaviours (e.g. glucose monitoring) |
| **Study design**   - Randomised controlled trials |

**Full text exclusions**

| **Author** | **Year** | **Title** | **Reason for exclusion** |
| --- | --- | --- | --- |
| Alothman | 2022 | Virtual Behavioral Intervention to Promote Healthy Lifestyle Behaviors: A Feasibility RCT during COVID-19 Pandemic. | Intervention not delivered by physical therapist |
| Anderson | 2014 | The impact of a bodyweight and physical activity intervention (BeWEL) initiated through a national colorectal cancer screening programme: randomised controlled trial. | Intervention not motivational interviewing (MI) |
| Ang | 2013 | Research to Encourage Exercise for Fibromyalgia (REEF): use of motivational interviewing, outcomes from a randomized-controlled trial | Intervention not delivered by physical therapist |
| Armstrong | 2021 | Behavioural modification interventions alongside pulmonary rehabilitation improve COPD patients' experiences of physical activity. | Intervention not MI |
| Armstrong | 2021 | A pilot rct assessing the inclusion of physical Activity counselling to standard care Pulmonary rehabilitation in patients with COPD | Not randomized controlled trial (RCT) |
| Aunger | 2020 | A novel behavioural INTErvention to REduce Sitting Time in older adults undergoing orthopaedic surgery (INTEREST): results of a randomised-controlled feasibility study | Intervention not delivered by physical therapist |
| Barker-Collo | 2015 | Improving adherence to secondary stroke prevention strategies through motivational interviewing: randomized controlled trial | Intervention not delivered by physical therapist |
| Barrett | 2018 | Integrated motivational interviewing and cognitive behaviour therapy can increase physical activity and improve health of adult ambulatory care patients in a regional hospital: the Healthy4U randomised controlled trial. | Intervention not MI |
| Barrett | 2022 | Physical activity telephone coaching intervention for insufficiently physically active ambulatory hospital patients: Economic evaluation of the Healthy 4U-2 randomised controlled trial | Intervention not MI |
| Befort | 2008 | Motivational interviewing fails to improve outcomes of a behavioral weight loss program for obese African American women: a pilot randomized trial. | Intervention not delivered by physical therapist |
| Bennett | 2007 | Motivational Interviewing to Increase Physical Activity in Long-Term Cancer Survivors: A Randomized Controlled Trial | Intervention not delivered by physical therapist |
| Bennett | 2008 | A telephone-only motivational intervention to increase physical activity in rural adults: a randomized controlled trial | Intervention not delivered by physical therapist |
| Benzo | 2019 | Effect of home pulmonary rehab plus health coaching on copd self-management: A randomized study | Not RCT |
| Benzo | 2016 | Health coaching and chronic obstructive pulmonary disease rehospitalization. A randomized study | Intervention not delivered by physical therapist |
| Blackford | 2016 | Effects of a home-based intervention on diet and physical activity behaviours for rural adults with or at risk of metabolic syndrome: a randomised controlled trial | Intervention not delivered by physical therapist |
| Bombardier | 2008 | The efficacy of telephone counseling for health promotion in people with multiple sclerosis: a randomized controlled trial. | Intervention not delivered by physical therapist |
| Bombardier | 2013 | Telephone-based physical activity counseling for major depression in people with multiple sclerosis | Intervention not delivered by physical therapist |
| Brodie | 2008 | Motivational interviewing to change quality of life for people with chronic heart failure: a randomised controlled trial. | Intervention not delivered by physical therapist |
| Brodie | 2005 | Motivational interviewing to promote physical activity for people with chronic heart failure. | Intervention not delivered by physical therapist |
| Brown | 2015 | A multicomponent behavioral intervention to reduce stroke risk factor behaviors: the stroke health and risk education cluster-randomized controlled trial | Intervention not delivered by physical therapist |
| Burgess | 2022 | Taking ACTION to Reduce Pain: a Randomized Clinical Trial of a Walking-Focused, Proactive Coaching Intervention for Black Patients with Chronic Musculoskeletal Pain. | Intervention not delivered by physical therapist |
| Campbell | 2009 | A randomized trial of tailoring and motivational interviewing to promote fruit and vegetable consumption for cancer prevention and control. | Intervention not delivered by physical therapist |
| Carels | 2007 | Using motivational interviewing as a supplement to obesity treatment: a stepped-care approach. | Intervention not delivered by physical therapist |
| Celano | 2020 | Feasibility and preliminary efficacy of a positive psychology-based intervention to promote health behaviors in heart failure: the REACH for health study | Intervention not delivered by physical therapist |
| Chair | 2012 | Short-term effect of motivational interviewing on clinical and psychological outcomes and health-related quality of life in cardiac rehabilitation patients with poor motivation in Hong Kong: a randomized controlled trial. | Intervention not delivered by physical therapist |
| Chen | 2012 | Effects of motivational interviewing intervention on self-management, psychological and glycemic outcomes in type 2 diabetes: a randomized controlled trial | Intervention not delivered by physical therapist |
| Chiang | 2019 | Motivational Counseling to Reduce Sedentary Behaviors and Depressive Symptoms and Improve Health-Related Quality of Life Among Women With Metabolic Syndrome. | Intervention not delivered by physical therapist |
| Collins | 2019 | Efficacy of a multi-component intervention to promote physical activity among Latino adults: a randomized controlled trial | Intervention not delivered by physical therapist |
| Connell | 2018 | Increasing physical activity and reducing sedentary behaviour in stroke survivors with the use of a personalised behavioural intervention: A feasibility study | Not RCT |
| de Blok | 2006 | The effects of a lifestyle physical activity counseling program with feedback of a pedometer during pulmonary rehabilitation in patients with COPD: A pilot study | Intervention not MI |
| de Vries | 2015 | Personalized physiotherapy in frail older adults with mobility problems is (cost)-effective in improving physical activity and frailty: A RCT | Not RCT |
| de Vries | 2016 | Patient-centred physical therapy is (cost-) effective in increasing physical activity and reducing frailty in older adults with mobility problems: a randomized controlled trial with 6 months follow-up. | Intervention not MI |
| DiMarco | 2009 | The use of motivational interviewing techniques to enhance the efficacy of guided self-help behavioral weight loss treatment | Intervention not delivered by physical therapist |
| Ehrlich-Jones | 2019 | Motivational Interviewing to Increase Physical Activity in Persons with Parkinson's Disease | Not RCT |
| English | 2016 | Reducing Sitting Time After Stroke: A Phase II Safety and Feasibility Randomized Controlled Trial. | Intervention not delivered by physical therapist |
| Everett | 2021 | A randomized controlled trial of motivational interviewing as a tool to enhance secondary prevention strategies in cardiovascular disease (MICIS study). | Intervention not delivered by physical therapist |
| Flores | 2020 | Effect of motivational interviewing on self-care of people with heart failure: a randomized clinical trial | Intervention not delivered by physical therapist |
| Friederichs | 2016 | Motivational interviewing and self-determination theory in a web-based computer tailored physical activity intervention: a randomized controlled trial | Intervention not delivered by physical therapist |
| Friederichs | 2015 | Long term effects of self-determination theory and motivational interviewing in a web-based physical activity intervention: randomized controlled trial | Intervention not delivered by physical therapist |
| Gilbert | 2018 | A randomized trial of a motivational interviewing intervention to increase lifestyle physical activity and improve self-reported function in adults with arthritis | Intervention not delivered by physical therapist |
| Gillham | 2010 | Impact of enhanced secondary prevention on health behaviour in patients following minor stroke and transient ischaemic attack: a randomized controlled trial. | Intervention not MI |
| Harland | 1999 | The Newcastle exercise project: a randomised controlled trial of methods to promote physical activity in primary care [with consumer summary] | Intervention not delivered by physical therapist |
| Holland | 2016 | Low cost home-based pulmonary rehabilitation for chronic obstructive pulmonary disease: A randomized controlled equivalence trial | Not RCT |
| Hollis | 2014 | Compliance to step count and vegetable serve recommendations mediates weight gain prevention in mid-age, premenopausal women. Findings of the 40-Something RCT | Intervention not delivered by physical therapist |
| Huang | 2018 | Effectiveness of self-management support in maintenance haemodialysis patients with hypertension: a pilot cluster randomized controlled trial | Intervention not delivered by physical therapist |
| Huber | 2015 | Telecoaching plus a portion control plate for weight care management: a randomized trial. | Intervention not delivered by physical therapist |
| Huffman | 2021 | A positive psychology-motivational interviewing program to promote physical activity in type 2 diabetes: the BEHOLD-16 pilot randomized trial | Intervention not delivered by physical therapist |
| Hyman | 2007 | Simultaneous versus sequential counseling for multiple behavior change | Intervention not delivered by physical therapist |
| Ismail | 2020 | Reducing weight and increasing physical activity in people at high risk of cardiovascular disease: a randomised controlled trial comparing the effectiveness of enhanced motivational interviewing intervention with usual care | Intervention not delivered by physical therapist |
| Janssen | 2014 | A self-regulation lifestyle program for post-cardiac rehabilitation patients has long-term effects on exercise adherence. | Intervention not delivered by physical therapist |
| Janssen | 2013 | Beyond resolutions? A randomized controlled trial of a self-regulation lifestyle programme for post-cardiac rehabilitation patients. | Intervention not delivered by physical therapist |
| Kaleth | 2018 | Obesity moderates the effects of motivational interviewing treatment outcomes in fibromyalgia | Intervention not delivered by physical therapist |
| Kanzawa-Lee | 2022 | Efficacy of the Motivational Interviewing-Walk Intervention for Chemotherapy-Induced Peripheral Neuropathy and Quality of Life During Oxaliplatin Treatment: A Pilot Randomized Controlled Trial. | Intervention not delivered by physical therapist |
| KatriMaria | 2021 | Effects of a home-based rehabilitation program in community-dwelling older people after discharge from hospital: a subgroup analysis of a randomized controlled trial [with consumer summary] | Intervention not MI |
| Keukenkamp | 2018 | An explorative study on the efficacy and feasibility of the use of motivational interviewing to improve footwear adherence in persons with diabetes at high risk for foot ulceration | Intervention not delivered by physical therapist |
| Khan | 2022 | Impact of patient and family engagement in improving continuous positive airway pressure adherence in patients with obstructive sleep apnea: a randomized controlled trial [with consumer summary] | Intervention not MI |
| Kim | 2017 | Sustained benefits of exercise-based motivational interviewing, but only among nonusers of opioids in patients with fibromyalgia | Intervention not delivered by physical therapist |
| Knittle | 2016 | Explaining physical activity maintenance after a theory-based intervention among patients with rheumatoid arthritis: process evaluation of a randomized controlled trial [with consumer summary] | Intervention not MI |
| Knittle | 2015 | Targeting motivation and self-regulation to increase physical activity among patients with rheumatoid arthritis: a randomised controlled trial | Intervention not MI |
| Kratz | 2014 | Affective mediators of a physical activity intervention for depression in multiple sclerosis | Intervention not delivered by physical therapist |
| Lai | 2014 | The efficacy of a brief motivational enhancement education program on continuous positive airway pressure adherence in obstructive sleep apnea: a randomized controlled trial | Intervention not delivered by physical therapist |
| Lilienthal | 2014 | Telephone-based motivational interviewing to promote physical activity and stage of change progression in older adults | Intervention not delivered by physical therapist |
| Lo | 2020 | Effects of Individualized Aerobic Exercise Training on Physical Activity and Health-Related Physical Fitness among Middle-Aged and Older Adults with Multimorbidity: A Randomized Controlled Trial. | Intervention not delivered by physical therapist |
| Mahmoodabad | 2017 | The effect of motivational interviewing-based intervention using self-determination theory on promotion of physical activity among women in reproductive age: a randomized clinical trial | Intervention not MI |
| Mangyo | 2020 | Intervention using behavior modification techniques to improve the lifestyle of high-risk metabolic syndrome patients. | Intervention not MI |
| Marcus | 2007 | Telephone versus print delivery of an individualized motivationally tailored physical activity intervention: project STRIDE | Intervention not delivered by physical therapist |
| Marques | 2017 | Efficacy of a randomized controlled self-regulation based physical activity intervention for chronic fatigue: mediation effects of physical activity progress and self-regulation skills [with consumer summary] | Intervention not delivered by physical therapist |
| Marques | 2015 | Effects of a self-regulation based physical activity program (the "4-STEPS") for unexplained chronic fatigue: a randomized controlled trial. | Intervention not delivered by physical therapist |
| Mayberry | 2014 | Adaptation, education, and motivation: improving evidence-based medication adherence among adults with type 2 diabetes | Not RCT |
| McMullen | 2009 | Motivational interviewing to increase exercise in multiple sclerosis | Not RCT |
| Mhurchu | 1998 | Randomized clinical trial comparing the effectiveness of two dietary interventions for patients with hyperlipidaemia | Participants not seeking physical therapy |
| Morowatisharifabad | 2021 | The Effect of Integrated Intervention Based on Protection Motivation Theory and Implementation Intention to Promote Physical Activity and Physiological Indicators of Patients with Type 2 Diabetes. | Intervention not MI |
| Movahedi | 2018 | Comparison of group motivational interviewing and multimedia education on elderly lifestyle | Intervention not MI |
| Murphy | 2012 | An evaluation of the effectiveness and cost effectiveness of the National Exercise Referral Scheme in Wales, UK: a randomised controlled trial of a public health policy initiative. | Intervention not delivered by physical therapist |
| Nooijen | 2016 | A behavioural intervention increases physical activity in people with subacute spinal cord injury: a randomised trial. | Intervention not delivered by physical therapist |
| Norweg | 2023 | Mind-Body Intervention for Dysfunctional Breathing in Chronic Obstructive Pulmonary Disease: Feasibility Study and Lessons Learned | Intervention not delivered by physical therapist |
| Nourizadeh | 2020 | The Effect of Motivational Interviewing on Women with Overweight and Obesity Before Conception. | Intervention not delivered by physical therapist |
| Ogedegbe | 2013 | The Counseling Older Adults to Control Hypertension (COACH) trial: design and methodology of a group-based lifestyle intervention for hypertensive minority older adults. | Intervention not delivered by physical therapist |
| Oldroyd | 2006 | Randomised controlled trial evaluating lifestyle interventions in people with impaired glucose tolerance. | Intervention not MI |
| Papandonatos | 2012 | Mediators of physical activity behavior change: findings from a 12-month randomized controlled trial | Intervention not MI |
| Prochaska | 2008 | Initial efficacy of MII, TTM tailoring and HRIs with multiple behaviors for employee health promotion | No measure of behaviour change |
| Quirk | 2012 | Pilot trial of motivational interviewing in patients with peripheral artery disease | Intervention not delivered by physical therapist |
| Reinhardt | 2012 | lmplementing lifestyle change through phone-based motivational interviewing in rural-based women with previous gestational diabetes mellitus | Intervention not delivered by physical therapist |
| Rouleau | 2017 | Results from a randomized feasibility trial of motivational interviewing to promote participation in outpatient cardiac rehabilitation | Not RCT |
| Rouleau | 2018 | The evaluation of a brief motivational intervention to promote intention to participate in cardiac rehabilitation: A randomized controlled trial. | Intervention not delivered by physical therapist |
| Schillebeeckx | 2021 | Behavioural modification interventions alongside pulmonary rehabilitation improve COPD patients' experiences of physical activity | Not RCT |
| Schorno | 2022 | Effectiveness of individual exercise and sport counseling based on motives and goals: a randomized controlled trial | Intervention not delivered by physical therapist |
| Seah | 2022 | Effectiveness of a Cluster Randomized Controlled Trial Involving Community-Based Intervention for Older Adults With Type 2 Diabetes Mellitus in Singapore | Intervention not delivered by physical therapist |
| Simpson | 2015 | A feasibility randomised controlled trial of a motivational interviewing-based intervention for weight loss maintenance in adults. | Intervention not delivered by physical therapist |
| Snoek | 2020 | Effectiveness of Home-Based Mobile Guided Cardiac Rehabilitation as Alternative Strategy for Nonparticipation in Clinic-Based Cardiac Rehabilitation Among Elderly Patients in Europe: A Randomized Clinical Trial | Intervention not MI |
| Soliman | 2013 | Motivational interviewing as educational program in improving cardiac risk factors control in patients post myocardial infarction | Not RCT |
| Swenson | 2006 | Physical activity in women receiving chemotherapy for breast cancer: patterns and adherence to an intervention protocol. | Not RCT |
| Swoboda | 2017 | Impact of a goal setting and decision support telephone coaching intervention on diet, psychosocial, and decision outcomes among people with type 2 diabetes. | Intervention not delivered by physical therapist |
| Ter Hoeve | 2018 | Effects of two behavioral cardiac rehabilitation interventions on physical activity: A randomized controlled trial. | Intervention not MI |
| Thomsen | 2016 | Motivational counselling and SMS-reminders for reduction of daily sitting time in patients with rheumatoid arthritis: a descriptive randomised controlled feasibility study. | Intervention not delivered by physical therapist |
| Thomsen | 2020 | Sustained Long-Term Efficacy of Motivational Counseling and Text Message Reminders on Daily Sitting Time in Patients With Rheumatoid Arthritis: Long-Term Follow-up of a Randomized, Parallel-Group Trial. | Intervention not delivered by physical therapist |
| Tse | 2013 | Motivational interviewing and exercise programme for community-dwelling older persons with chronic pain: a randomised controlled study | Intervention not MI |
| Tsianakas | 2017 | CanWalk: a feasibility study with embedded randomised controlled trial pilot of a walking intervention for people with recurrent or metastatic cancer [with consumer summary] | Intervention not delivered by physical therapist |
| Turner | 2016 | Improving fatigue and depression in individuals with multiple sclerosis using telephone-administered physical activity counseling. | Intervention not delivered by physical therapist |
| Turner | 2014 | Telephone counseling and home telehealth monitoring to improve medication adherence: results of a pilot trial among individuals with multiple sclerosis | Intervention not delivered by physical therapist |
| Turunen | 2020 | Effects of an individually targeted multicomponent counseling and home-based rehabilitation program on physical activity and mobility in community-dwelling older people after discharge from hospital: a randomized controlled trial | Intervention not MI |
| van Beers | 2018 | Clinical outcome and cost-effectiveness of 1-year nutritional intervention program in COPD | Not RCT |
| van Beers | 2020 | Clinical outcome and cost-effectiveness of a 1-year nutritional intervention programme in COPD patients with low muscle mass: The randomized controlled NUTRAIN trial. | Intervention not MI |
| van Keulen | 2011 | Tailored print communication and telephone motivational interviewing are equally successful in improving multiple lifestyle behaviors in a randomized controlled trial | Intervention not delivered by physical therapist |
| van Keulen | 2021 | A randomized controlled trial comparing community lifestyle interventions to improve adherence to diet and physical activity recommendations: the VitalUM study | Intervention not delivered by physical therapist |
| Vellone | 2020 | Motivational interviewing to improve self-care in heart failure patients (MOTIVATE-HF): a randomized controlled trial | Intervention not delivered by physical therapist |
| Vlaar | 2017 | Effectiveness of a targeted lifestyle intervention in primary care on diet and physical activity among South Asians at risk for diabetes: 2-year results of a randomised controlled trial in the Netherlands [with consumer summary] | Intervention not delivered by physical therapist |
| Young | 2019 | Telephone-based motivational interviewing versus usual care in primary care to increase physical activity: a randomized pilot study | Intervention not delivered by physical therapist |

Participants not seeking physical therapy (n = 1)

Not RCT (n = 13)

No measure of behaviour change (n = 1)

Intervention not MI (n = 22)

Intervention not delivered by physical therapist (n = 71)

**PEDro methodological quality assessment**

| Study | Eligibility criteria specified | Random allocation | Concealed allocation | Groups similar at baseline | Participant blinding | Therapist blinding | Assessor blinding | < 15% dropouts | Intention-to-treat analysis | Between-group difference reported | Point estimate and variability reported | TOTAL  /10 |
| --- | --- | --- | --- | --- | --- | --- | --- | --- | --- | --- | --- | --- |
| Arbillaga-Etxarri 2018 | Y | Y | N | Y | N | N | Y | N | Y | Y | Y | 6 |
| Arkkukangas 2019  and Tuvemo Johnson 2021 | Y | Y | Y | Y | N | N | Y | Y | Y | Y | Y | 8 |
| Burtin 2015 | Y | Y | Y | Y | N | N | Y | N | N | Y | Y | 6 |
| Dennett 2018 | Y | Y | Y | Y | N | N | Y | Y | Y | Y | Y | 8 |
| Larsen 2021 | Y | Y | Y | Y | N | N | Y | Y | Y | Y | Y | 8 |
| O'Halloran 2016 | Y | Y | Y | Y | N | N | Y | N | N | Y | Y | 6 |
| Rausch Osthoff 2021 | Y | Y | Y | Y | N | N | Y | N | N | Y | Y | 6 |
| Reid 2012 | Y | Y | Y | Y | N | N | Y | N | Y | Y | Y | 7 |
| Pellegrini 2022 | Y | Y | N | Y | N | N | N | Y | N | Y | Y | 5 |
